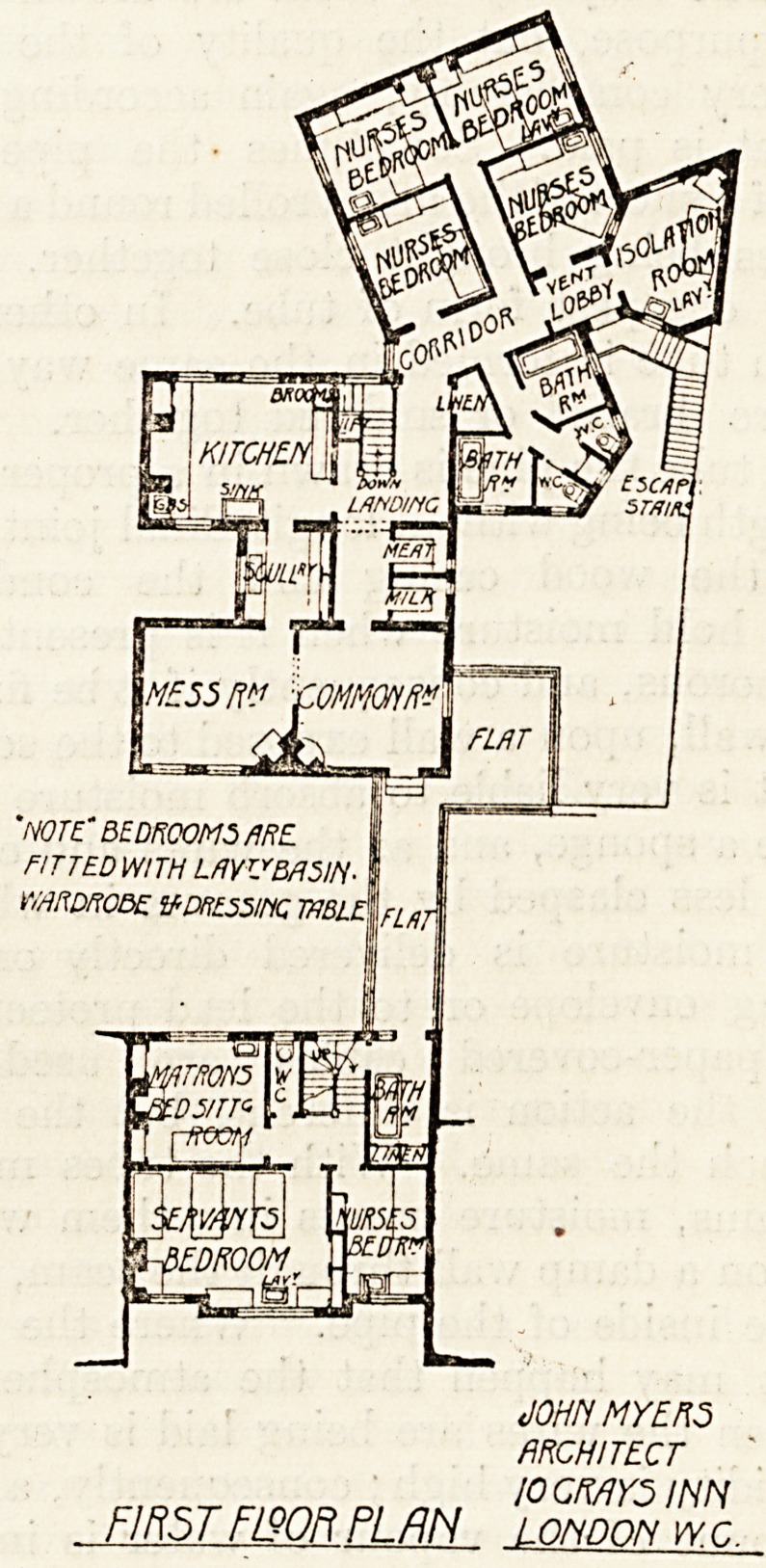# The Sick Room Helps Society's New Maternity Home

**Published:** 1912-02-24

**Authors:** 


### HOSPITAL ARCHITECTURE AND CONSTRUCTION.

**[Communications on this subject should be marked "Architecture" in the left-hand top corner of the envelope.]**

### The Sick Room Helps Society's New Maternity Home.

THIS very interesting little building, besides being the headquarters of the Sick Room Helps Society, of Underwood Street, Mile End New Town, contains a complete Maternity Home and a Home for Nurses.

"Sick-room helps" are nurses who have received

GROUND FLOOR PLAN

a certain amount of training and who visit the homes of the sick poor, attend to the invalid, wash the children, cook the dinner and generally help in any way they can. Some seventy of these nurses are employed by the Society, and the front part of the new building contains, on the ground floor, the offices for the control and organisation of the work. On the upper floor is sleeping accommodation for the Matron, three servants, and one nurse.

The Maternity Home is arranged on the ground floor of the back building and contains a ward for four beds, two wards for one bed each, a labour room, two nurses' bedrooms, a bathroom, ward, scullery, and sanitary offices. On the upper floor are the kitchen offices, mess room and common room for nurses, an isolation room, four bedrooms for nurses (one of which is attached to the isolation room), two bathrooms and two w.c.s. An outside staircase from this floor provides means of escape in case of fire, and also separate access from outside to the isolation room.

The site is not spacious, and the access of light and air to the wards and adjacent rooms is apparently restricted by the two small yards. Whether this is sufficient depends on the immediate surroundings, and if there are open yards on all three sides there should be no lack of either.

The planning of the buildings shows no little skill

FIRST FLOOR PLAN

in dealing with a difficult site. The architect is Mr. John Myers.

## Figures and Tables

**Figure f1:**
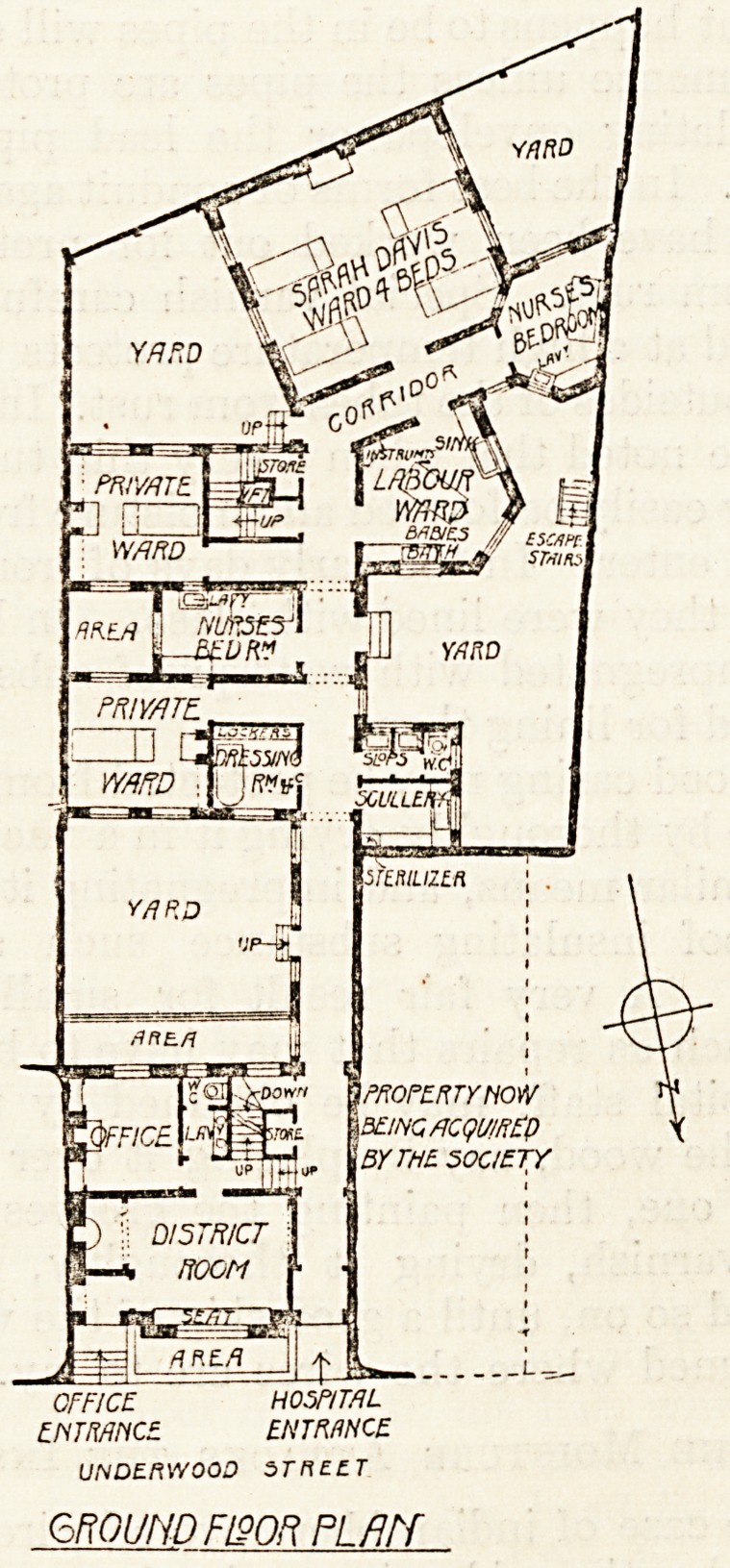


**Figure f2:**